# A structural approach to the graceful coloring of a subclass of trees

**DOI:** 10.1016/j.heliyon.2023.e19563

**Published:** 2023-09-01

**Authors:** Laavanya D, Devi Yamini S

**Affiliations:** Vellore Institute of Technology, Chennai, Tamil Nadu, India

**Keywords:** Graceful coloring, Graceful chromatic number, Coloring algorithm, Trees

## Abstract

Let M={1,2,..m} and *G* be a simple graph. A graceful *m*-coloring of *G* is a proper vertex coloring of G using the colors in *M* which leads to a proper edge coloring using M∖{m} colors such that the associated color of each edge is the absolute difference between their end vertices. The graceful chromatic number $\chi_{g}(G)=\text{ min }\{m:G\text{ admits a graceful}\;\text{m}\text{− coloring }\}$. We prove that $5\leq \chi_{g}(T)\leq 7$, where *T* is a tree with Δ=4. Furthermore, we categorize the trees into three types along with its characterization and the related coloring algorithm are presented in this study.

## 1 Introduction

In 1967, Alexander Rosa [8] defined graph labeling as follows: A label from {0,1,2,...} is given to every vertex or edge (or both) of a graph G=(V,E) satisfying certain conditions. Refer [5] for more details on graph labeling. Graceful labeling, a variant of graph labeling, was initially referred as *β*-labeling by Alexander Rosa [8]. Let L={0,1,...,|E(G)|}. A one-to-one function *ℓ* is a graceful labeling from the vertex set of *G* to *L* resulting in another one-to-one function $\ell^{⁎}$ from the edges of *G* to L∖{0}, where the label for every edge is the absolute difference between its end vertices.

The development in the field of graph theory led to the concept of graph coloring in which the vertex coloring and the edge coloring are the major topics of interest. A proper vertex coloring is assigning colors to the vertices of a graph *G* in such a way that different colors are assigned for adjacent vertices. In a similar manner, proper edge coloring is defined [2]. Chromatic number (χ(G)) is the least number of colors required for proper coloring the vertices of a graph, where as the chromatic index $\left(\chi^{\prime }(G)\right)$ is the least number of colors needed for proper coloring the edges of a graph. Graph coloring has extensive applications in various fields ([2], [6], [9]).

Graceful coloring is an extension of graceful labeling [3] in which both vertex and edge colorings are involved. For any two positive integers r,s with r<s, we define [r,s]={r,r+1,...,s−1,s}. A graceful *m*-coloring of *G* is a proper vertex coloring *ℓ* of *G* using the colors in M=[1,m], where m≥2, which leads to a proper edge coloring $\ell^{⁎}$ using M∖{m} colors such that the associated color of each edge is the absolute difference between their end vertices. The graceful chromatic number $\chi_{g}(G)=\text{ min }\{m:G\text{ admits graceful}\;\text{m}\text{− coloring }\}$.

For a subgraph *F* of *G*, $\chi_{g}(F)\leq \chi_{g}(G)$ and $\chi_{g}(G)\geq \Delta +1$, where Δ represents the maximum degree of *G* [3].

The Table 1 provides the existing results in graceful coloring. The graceful coloring of a few subclasses of unicyclic graphs [1] and a few variants of ladder graphs [7] are discussed in the literature. The Lemma 1.1 is useful in proving the main results of our paper, which was already proved in [7].

Lemma 1.1 *If*[1,Δ+i]*,*$i\in Z^{+}$*colors are used in graceful coloring of a graph G, then the vertex of maximum degree will receive the first and last i colors from*[1,Δ+i]*.*

Graceful coloring for many graph classes like bipartite graphs, complete graphs, trees, etc. are still open. Hence we focus on a subclass of trees.

**Table 1:** Existing results on *χ*_*g*_(*G*).

Graph *G*	*χ*_*g*_(*G*)	Bound for *χ*_*g*_(*G*)	Reference
Diameter at most 2	-	Lower bound: |*V*(*G*)|	[3]

*r*− regular graph	-	Lower bound: *r* + 2	[3]

Cycle *C*_*n*_, *n* ≥ 4	$\left\{ \begin{array}{cc} 4, & \text{if }n\neq 5\\ 5, & \text{if }n=5 \end{array}\right.\;$	-	[3]

Path *P*_*n*_, *n* ≥ 5	5	-	[3]

Wheel *W*_*n*_, *n* ≥ 6	*n*	-	[3]

Complete bipartite graph of order 2	*n*	-	[3]

Trees with maximum degree Δ	-	Upper bound: $\left\lceil \frac{5\Delta }{3}\right\rceil$	[3]

Caterpillar with a maximum degree vertex adjacent to two vertices of maximum degree	Δ + 2	-	[3]

Rooted trees of height 2	$\left\lceil \frac{1}{2}(3\Delta +1)\right\rceil$	-	[4]

Rooted trees of height 3	$\left\lceil \frac{1}{8}(13\Delta +1)\right\rceil$	-	[4]

Rooted trees of height 4	$\left\lceil \frac{1}{32}(53\Delta +1)\right\rceil$	-	[4]

Rooted trees with height at least $2+\lfloor \frac{1}{3}\Delta \rfloor$	$\left\lceil \frac{5}{3}\Delta \right\rceil$	-	[4]

**Our contributions:**

For the trees with maximum degree 4, we

– find the bound for the graceful chromatic number.
– give a structural characterization.
– provide an algorithm for graceful coloring.

## 2 Notations and terminologies

**Figure 1:**
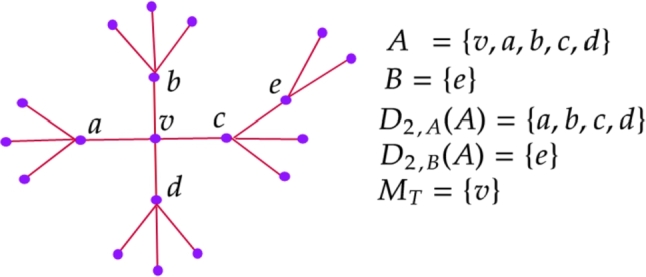
Illustration of notations.

Let T represent the class of trees *T* with maximum degree 4 and the following notations hold for all T∈T which is clearly illustrated in the Fig. 1.

⁎ d(v): degree of the vertex *v* in *T*
⁎ $d(v_{1},v_{2})$: distance between $v_{1},v_{2}$ in *T*
⁎ T[S]: induced subgraph of *T* on *S*
⁎ T(v): a tree in T with a root vertex *v* (a vertex of degree 4)
⁎ N(v): neighbors of *v* in *T*
⁎ A={v:d(v)=4}
⁎ B={v:d(v)=3}
⁎ For any X,S⊆V(T) and n∈N
  $D_{n,X}(S)=\{v\in X$: d(v,s)=n, for some s∈S}
⁎ $M_{T}=\{v\in A:N(v)\subseteq A\}$

## 3 Main results

It is proved that for any connected graph *G*, $\chi_{g}(G)\geq \Delta +1$ and for a nontrivial tree *T*, $\chi_{g}(T)\leq \lceil \frac{5\Delta }{3}\rceil$ where Δ is the maximum degree of the graph [3]. Hence, we conclude that, $5\leq \chi_{g}(T)\leq 7$ for all T∈T. In addition, we categorize T into three types based on the graceful chromatic number.

– Type I: Class of all trees T∈T such that $\chi_{g}(T)=5$
– Type II: Class of all trees T∈T such that $\chi_{g}(T)=6$
– Type III: Class of all trees T∈T such that $\chi_{g}(T)=7$

Lemma 3.1 *If a tree*T∈T*is gracefully colored using*[1,5]*, then*

(i) ℓ(v)∉[2,4]*, for*v∈A*of T*
(ii) ℓ(v)≠3*, for*v∈B*of T*

Proof For any v∈V(T), we denote N(v) as $\left\{v_{i}\right\}$, i∈[1,d(v)].

(i) The result is obvious from the Lemma 1.1.

(ii) Let v∈B. Suppose on the contrary, if ℓ(v)=3 then there exist $v_{i},v_{j}\in N(v)$ such that $\ell^{⁎}(vv_{i})=\ell^{⁎}(vv_{j})=(1$ or 2), for i,j∈[1,3] and i≠j. This leads to a contradiction to the fact of proper edge coloring. Hence ℓ(v)≠3. □

Proposition 3.2 *For any tree* T∈T *,* $\chi_{g}(T)\neq 5$ *if T satisfies one of the following conditions:*

*a)*T[A]*has a component which forms a path of length at least* 2*.*

*b)* N(v)⊆A∪B,wherev∈A *.*

*c)* N(v)⊆A,wherev∈B *.*

*d)* $|D_{2,v}(A)|\geq 3\;\text{(violating the condition (c))}\;$ *,* wherev∈A *.*

*e)*|N(v)∩(A∪B)|=2*or* 3 *and* $|D_{2,v}(A)|\leq 2$*,* wherev∈A*.*

Proof a) Note that for any v∈A, ℓ(v)∉[2,4] (by the Lemma 3.1). Suppose T[A] contain a component which forms a path of length k≥2, then the colors {1,5} are not sufficient for the graceful coloring of T[A], since $\chi_{g}(P_{n})\geq 3,\text{ for }n\geq 3$ [3].

b) Assume *v* as a root such that N(v)⊆A∪B which does not satisfy the condition (*a*). Then either d(N(v))=3 or there is exactly one neighbor of *v* with degree 4. Either of the case does not lead to graceful coloring using [1,5] (by the Lemma 3.1).

c) Let v∈B and N(v)={a,b,c}. Then ℓ(a)∪ℓ(b)∪ℓ(c)∈{1,5}, since d(a,b)=d(a,c)=d(b,c)=2. By a result in [3], it does not lead to the graceful coloring using [1,5].

d) Let $N(v)=\{v_{1},v_{2},v_{3},v_{4}\}$, where v∈A. Then there exist a,b,c∈A such that d(v,a)=d(v,b)=d(v,c)=2. Without loss of generality, assume that $v_{1}a,v_{2}b,v_{3}c\in E(T)$. Note that ℓ(v)∈{1,5} (by the Lemma 3.1). Suppose ℓ(v)=1. Then ℓ(a)=ℓ(b)=ℓ(c)=5. Clearly, $v_{1},v_{2}\text{ and }v_{3}$ cannot be colored with 3. Hence $\ell (v_{i})\in \{2,4\},1\leq i\leq 3$, which is not sufficient for graceful coloring using 5 colors.

e) Let v∈A with $N(v)=\{v_{1},v_{2},v_{3},v_{4}\}$. Let $a,b\in D_{2,v}(A)$, then d(v,a)=d(v,b)=2. Without loss of generality, assume $v_{1}a,v_{2}b\in E(T)$. Let ℓ(v)=1, then ℓ(a)=ℓ(b)=5. So $v_{1}$ and $v_{2}$ cannot be colored with 3. Observe that, $d(v_{3})\cup d(v_{4})\in \{3,4\}$. So $\ell (v_{3})\cup \ell (v_{4})\neq 3$ (by the Lemma 3.1). Clearly one of the neighbors of *v* is left uncolored while using [1,5] colors. The same argument holds when $|D_{2,v}(A)|=1$. □

The class of all trees in T which contain an induced subgraph with at least one of the condition stated in the Proposition 3.2 are denoted as $T_{1}$. Next, we construct another class of trees $T_{2}$ in T which do not contain any trees in $T_{1}$ as an induced subgraph.

Let *v* be a root containing a neighbor *u* whose degree is 1. We now state few conditions to generate trees in $T_{2}$:

$c_{1}$: N(v)∖u⊆A∪B
$c_{2}$: $|D_{2,v}(A)|\leq 2$ with |(N(v)∖u)∩(A∪B)|≤2
$c_{3}$: $|D_{2,v}(A)|=2$ and $|D_{2,v}(B)|=1$

Note that the trees that belong to T satisfying $c_{i}$, 1≤i≤3 do not belong to $T_{1}$.

Lemma 3.3 *If the trees*T∈T*satisfying the condition*$c_{1}$*or*$c_{2}$*or*$c_{3}$*are gracefully colored using*[1,5]*, then*

(i) $\ell (u_{1})=\ell (u_{2})=3$
(ii) $\ell (u_{3})=3\;\text{or}\;5$

*where*$u_{i}$*is the pendant vertex of the tree T satisfying the condition*$c_{i}$*,*i∈{1,2,3}*.*

Proof (i) If T(v) satisfies the condition $c_{1}$ with $N(v)=\{v_{1},v_{2},v_{3},u_{1}\}$, then ℓ(v)=1 or 5 (by the Lemma 3.1). Without loss of generality, assume ℓ(v)=1. By the condition $c_{1}$, $\ell (v_{1})\cup \ell (v_{2})\cup \ell (v_{3})\neq 3$ (by the Lemma 3.1). So, $\ell (u_{1})=3$ for a proper vertex coloring.

Now, we show that $\ell (u_{2})=3$ with two cases for a proper vertex coloring of *T*. Let T(v) satisfy the condition $\left(c_{2}\right)$ with $N(v)=\{v_{1},v_{2},v_{3},u_{2}\}$. It is clear that ℓ(v)∈{1,5} (by the Lemma 3.1). Without loss of generality, ℓ(v)=1.

**Case 1:** By the condition $c_{2}$, if there exists two neighbors of *v*, namely $v_{1}$ and $v_{2}$ with degree at least 3, then the colors for the vertices $v_{1}$ and $v_{2}$ cannot be 3 (by the Lemma 3.1). Also, by the condition $c_{2}$, there exists a vertex $a\in D_{2,v}(A)$ such that d(a,v)=2 with an edge $av_{3}$ in *T*. Thus ℓ(a)=5 and hence $\ell (v_{3})\neq 3$, in order to preserve the proper edge coloring of *T*. So, $\ell (u_{2})=3$.

**Case 2:** By the condition $c_{2}$, if there exists a neighbor of *v*, say $v_{1}$ with degree at least 3, then $\ell (v_{1})\neq 3$ (by the Lemma 3.1). Also, there exist two vertices $a,b\in D_{2,v}(A)$ such that d(a,v)=d(b,v)=2 with $av_{2}$, $av_{3}\in E(T)$. Since ℓ(v)=1, ℓ(a)=ℓ(b)=5. Clearly, $\ell (v_{2})$ or $(\ell (v_{3}))=3$ violates the proper edge coloring. So, $\ell (v_{2})\neq \ell (v_{3})\in \{2,4\}$. Hence, $\ell (u_{2})=3$.

(ii) For the tree T(v) satisfying the condition $c_{3}$, let $N(v)=\{v_{1},v_{2},v_{3}$, $u_{3}\}$ and ℓ(v)=1. Since $|D_{2,v}(A)|=2$, let $a,b\in D_{2,v}(A)$ with the corresponding paths $a-v_{1}-v$ and $b-v_{2}-v$. Clearly, ℓ(a)=ℓ(b)=5. So $\ell (v_{2})\neq \ell (v_{3})\in \{2,4\}$. Since $|D_{2,v}(B)|=1$, let $c\in D_{2,v}(B)$ with the corresponding path $c-v_{3}-v$ (by the condition $c_{3}$). So, it is clear that the color for the vertices $v_{3}$ and $u_{3}$ are 3 or 5. Thus, $\ell (u_{3})=3\text{ or }5$.

The same argument holds when ℓ(v)=5. □

Let us denote $A_{1}$ as the trees in T which satisfy either the condition $\left(c_{1}\right)$ or $\left(c_{2}\right)$ or both. Let $T_{0}$ be a tree that satisfy the condition $\left(c_{3}\right)$. We now define a new graph operation **‘‘Pendant path connect (**P**)’’**.

P: Let $G_{1}$ and $G_{2}$ be two vertex disjoint graphs with pendant vertices $u_{1}$ and $u_{2}$ respectively. This graph operation creates a new graph by adding a path $P_{n}(n\geq 2)$ from $u_{1}$ to $u_{2}$ (inclusive).

(P1): Apply the graph operation P for any two trees in $A_{1}$ by a path of length at most 4 excluding $P_{4}$, namely $u_{1}-u_{2}$, $u_{1}-a-u_{2}$ and $u_{1}-a-b-c-u_{2}$ such that d(a) and d(c) are not exactly 4.

(P2): Apply the graph operation P for any two trees in $A_{1}$ by an odd path $P_{n}(n\geq 7)$ as $u_{1}-a-b_{1}-b_{2}-b-3-...b_{k-1}-b_{k}-c-u_{2}$ with one of the following degree sequences in the order of the path.

– (2,2,4,2,4,...,2,4,2,2)
– (2,3,4,2,4,...,2,4,3,2)
– (2,2,4,2,4,...,2,4,3,2)
– (2,3,4,2,4,...,2,4,2,2)
– (2,2,4,3,4,...,3,4,2,2)
– (2,3,4,3,4,...,3,4,3,2)
– (2,2,4,3,4,...,3,4,3,2)
– (2,3,4,3,4,...,3,4,2,2)

(P3): Apply the graph operation P for a tree in $A_{1}$ or $T_{0}$ with $T_{0}$ by an edge joining $u_{1}$ and $u_{2}$.

Let $T_{2}$ be the class of all trees in T which contains the graph in $A_{1}$ or $T_{0}$ as an induced subgraph.

Proposition 3.4 $\chi_{g}(T)\neq 5$ *, for all* $T\in T_{2}$ *.*

Proof Let $T\in T_{2}$. Then there exist trees $T_{1}(u)$ and $T_{2}(v)$ with $N(u)=\{u_{1},u_{2},u_{3},u_{4}\}$ and $N(v)=\{v_{1},v_{2},v_{3},v_{4}\}$ such that $d(u_{1})=d(v_{1})=1$ before applying the graph operation P on $T_{1}(u)$ and $T_{2}(v)$. It is clear from the Lemma 3.1 that the colors for the root vertices *u* and *v* are 1 or 5.

**Case 1:** If $T\in T_{2}$ is constructed using (P1), then from the Lemma 3.3, $\ell (u_{1})=\ell (v_{1})=3$. Hence the path connecting $u_{1}$ and $v_{1}$ in *T* cannot be $P_{2}$ or $P_{3}$. If $u_{1}$ and $v_{1}$ are connected using the path $P_{5}$, say $u_{1}-a-b-c-v_{1}$. Then ℓ(a)≠ℓ(c)∉{1,3,5}. Hence ℓ(a)≠ℓ(c)∈{2,4}. Without loss of generality, ℓ(a)=2 and ℓ(c)=4. Then the only possible choice of color for the vertex *b* is 1 or 5 which will not induce a proper edge coloring. Hence, the colors [1,5] are not sufficient for graceful coloring of the trees in $T_{2}$ constructed from (P1).

**Case 2:** If $T\in T_{2}$ is constructed using (P2), then from the Lemma 3.3, $\ell (u_{1})=\ell (v_{1})=3$. This case is an extension of the path in (P1) to (Pn) for odd values of n≥7. Consider the path $P_{5}$ mentioned in Case 1 as $u_{1}-a-b-c-v_{1}$, preserving the graph properties of $u_{1}$ and $v_{1}$. We now replace the vertex *b* in the path by $b_{1},b_{2},...,b_{n-4}$. Consider one of the degree sequences mentioned in (P2) in the order of the path $u_{1}-a-b_{1}-b_{2}-b_{3}-...-b_{n-5}-b_{n-4}-c-v_{1}$. Without loss of generality, let ℓ(u)=1 then $\ell (u_{1})=\ell (v_{1})=3$ (by the Lemma 3.3). Also, from the above Case 1, ℓ(a)∈{2,4}. So, we have two subcases with respect to ℓ(a).

Subcase 1: If ℓ(a)=2 then the vertices $b_{1},b_{3},b_{5},...,b_{n-4}$ will receive the colors 1 and 5 alternatively (vertices of degree 4). Note that $\ell (b_{1})\neq 1$, (violates a proper edge coloring). Hence $\ell (b_{1})=5$ and

$$\ell (b_{i})=\left\{ \begin{array}{cc} 1, & \text{if }i\equiv 3(\text{mod }4)\\ 5, & \text{if }i\equiv 5(\text{mod }4) \end{array}\right.$$

Similarly, the vertices $b_{2},b_{4},b_{6},...,b_{n-5}$ will receive the colors 2 and 4 alternatively. Observe that $\ell (b_{2})=4$ (since ℓ(a)=2). Hence

$$\ell (b_{i})=\left\{ \begin{array}{cc} 4, & \text{if }i\equiv 2(\text{mod }4)\\ 2, & \text{if }i\equiv 4(\text{mod }4) \end{array}\right.$$

Further, ℓ(c)∈{2,4} violates a proper coloring. Hence, the colors [1,5] are not sufficient for the graceful coloring of the trees $T_{2}$ constructed from Step 2.

Subcase 2: If ℓ(a)=4, then $\ell (b_{1})\neq 5$ (since $\ell (u_{1})=3$). Hence, $\ell (b_{1})=1$ and

$$\ell (b_{i})=\left\{ \begin{array}{cc} 1, & \text{if }i\equiv 5(\text{mod }4)\\ 5, & \text{if }i\equiv 3(\text{mod }4) \end{array}\right.$$

Similarly, $\ell (b_{2})=2$ and

$$\ell (b_{i})=\left\{ \begin{array}{cc} 4, & \text{if }i\equiv 4(\text{mod }4)\\ 2, & \text{if }i\equiv 2(\text{mod }4) \end{array}\right.$$

Also ℓ(c)∈{2,4} violates a proper coloring. The same argument will hold if ℓ(u)=5. Hence, the colors [1,5] are not sufficient for graceful coloring of the trees $T_{2}$ constructed from (P2).

**Case 3:** If $T\in T_{2}$ is constructed using (P3) by an edge joining $u_{1}$ and $v_{1}$, then ℓ(u)∪ℓ(v)∈{1,5}. If *T* is obtained by taking two copies of $T_{0}$ using the operation P, then $\ell (u_{1})\neq \ell (v_{1})\in \{5,3\}$ (by the Lemma 3.3). Obviously this violates a proper coloring. If *T* is obtained from two trees, one from $A_{1}$ with the tree $T_{0}$ having roots *u* and *v* respectively, then by the Lemma 3.3, $\ell (v_{1})\in \{3,5\}$. Since $\ell (u_{1})=3$ (by the Lemma 3.3), $\ell (v_{1})=5$. Also, ℓ(u)∪ℓ(v)∈{1,5} will not induce a proper edge coloring. Hence, the colors [1,5] are not sufficient for graceful coloring of the trees $T_{2}$ constructed from (P3).

Thus, in all Cases $\chi_{g}(T)\neq 5$, for all $T\in T_{2}$. □

Denote $T_{1}$ as a tree in T containing a path a−b−c with d(a)=d(c)=4 and d(b)=3 such that N(b)={a,c,u} and d(u)=1. Also, denote $T_{2}$ as a tree in T containing a path a−b−c with d(a)=d(b)=4 and d(c)=3 such that N(c)={b,d,u} and d(u)=1. We now define a new graph operation **‘‘Pendant vertex identification (**V**)’’**.

V: Consider two or more vertex disjoint graphs with at least one pendant vertex in each of the graphs. This graph operation merges the pendant vertices in each graph into a single vertex, preserving their adjacencies. We now construct $T_{3}$ as the class of all trees in T which contain an induced subgraph by using (A),(B) and (*C*).

**(A)** Applying the graph operation V for $T_{1}$ with a tree from $A_{1}$ or the tree $T_{0}$.

**(B)** Applying the graph operation V for $T_{1}$ and $T_{2}$.

**(C)** Repeated application of V on $T_{1}$.

Proposition 3.5 $\chi_{g}(T)\neq 5$ *, for all* $T\in T_{3}$ *.*

Proof Suppose on contrary, $\chi_{g}(T)=5$, for all $T\in T_{3}$.

**Case 1:** If $T\in T_{3}$ is constructed using (A), then $u_{1}$ and $v_{1}$ are the pendant vertices of the tree belonging to $A_{1}\cup A_{2}$ and $T_{1}$ respectively before applying the graph operation V. Also, $\ell (u_{1})=3$ or 5 (by the Lemma 3.3). By the construction of $T_{1}$, there exists a vertex b∈B with $N(b)=\{a,c,v_{1}\}$, such that d(a)=d(c)=4. Hence ℓ(a)≠ℓ(c)∈{1,5}. Thus, $\ell (b)\cap \ell (v_{1})\notin \{1,3,5\}$ (since $d(a,v_{1})=d(c,v_{1})=2$). This leads to a contradiction that the pendant vertex $u_{1}$ cannot be identified with the pendant vertex $v_{1}$ for the graceful coloring using [1,5]. Hence, $\chi_{g}(T)\neq 5$.

**Case 2:** If $T\in T_{3}$ is constructed using (B), then there exist a vertex u∈B in $T_{1}$ with $N(u)=\{x,u_{2},u_{3}\}$ such that $d(u_{2})=d(u_{3})=4$ and there exists a path a−b−c in $T_{2}$ with d(a)=d(b)=4, d(c)=3 such that $N(c)=\{b,x,v_{2}\}$. Then $\ell (u_{2})\neq \ell (u_{3})\in \{1,5\}$ and ℓ(u)∩ℓ(x)∉{1,3,5} (by the Lemma 3.1). Also, ℓ(a)≠ℓ(b)∈{1,5} and ℓ(c)∉{1,3,5} (by the Lemma 3.1). Thus, there are only two colors {2,4} for the three vertices u,x and *c*, which does not induce a proper coloring [1,5], which leads to a contradiction. Hence, $\chi_{g}(T)\neq 5$.

**Case 3:** If $T\in T_{3}$ is constructed using (C), then there exist a,b∈B with $N(a)=\{a_{1},a_{2},x\}$ and $N(b)=\{b_{1},b_{2},x\}$. Let $d(a_{1})=d(a_{2})=d(b_{1})=d(b_{2})=4$, then $\ell (a_{1})\neq \ell (a_{2})\in \{1,5\}$ and $\ell (b_{1})\neq \ell (b_{2})\in \{1,5\}$. Also, ℓ(a)≠ℓ(b)∈{2,4}. Thus, the only choice of color for the vertex *x* is 3, which will not induce a proper edge coloring, a contradiction. Hence, $\chi_{g}(T)\neq 5$. □

Denote $T_{3}$ as a tree in T with root *v* such that N(v)={a,b,c,u}, where {a,b,c}∈A and d(u)=1.

**Construction of**${T_{3}}^{\prime }$: Applying the graph operation V on three copies of $T_{3}$.

Clearly the degree of the identified vertex *v* in ${T_{3}}^{\prime }$ is 3 and N(v)⊆A. So, ${T_{3}}^{\prime }$ belongs to $T_{1}$. Hence by the Proposition 3.2, $\chi_{g}({T_{3}}^{\prime })\neq 5$.

Remark 3.6 If T∈T is gracefully colored using [1,6], then ℓ(v)∉{3,4}, where v∈A.

Proposition 3.7 $\chi_{g}({T_{3}}^{\prime })\neq 6$*.*

Proof Suppose on contrary, $\chi_{g}({T_{3}}^{\prime })=6$. From the construction of ${T_{3}}^{\prime }$, it is clear that, there exist an identified vertex *v* with degree 3, such that N(v)⊆A. Let N(v)={a,b,c}, $N(a)=\{a_{1},a_{2},a_{3},v\}$, $N(b)=\{b_{1},b_{2},b_{3},v\}$ and $N(c)=\{c_{1},c_{2},c_{3},v\}$. It is to be noted that, all the neighbors of a,b and *c* except *v* are in *A*. Hence, ℓ(a)∩ℓ(b)∩ℓ(c)∈{1,2,5,6} (by the Lemma 3.3). Without loss of generality, let ℓ(a)=1, ℓ(b)=2 and ℓ(c)=5, then $\ell (a_{1})\cap \ell (a_{2})\cap \ell (a_{3})\in \{2,5,6\}$, $\ell (b_{1})\cap \ell (b_{2})\cap \ell (b_{3})\in \{1,5,6\}$ and $\ell (c_{1})\cap \ell (c_{2})\cap \ell (c_{3})\in \{1,2,6\}$. Thus, ℓ(v)=3 or 4. If ℓ(v)=3, then $\ell^{⁎}(av)=\ell^{⁎}(vc)=2$ which is not possible. Similarly, if ℓ(v)=4, then $\ell^{⁎}(vc)=\ell^{⁎}(cc_{i})=1$, for 1≤i≤3 which is not possible. Hence a contradiction. Similar argument holds for all possible combination of colors for ℓ(a)∩ℓ(b)∩ℓ(c)∈{1,2,5,6}. Hence $\chi_{g}({T_{3}}^{\prime })\neq 6$. □

### 3.1 Type-I trees

Let Type I be the class of all trees in $T\setminus (T_{1}\cup T_{2}\cup T_{3})$.

Theorem 3.8 *For a tree*T∈T*,*$\chi_{g}(T)=5$*if and only if T is of Type I.*

**Figure 2:**
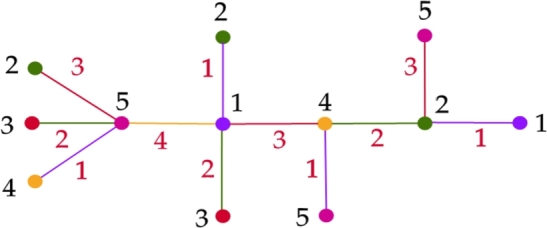
Graceful 5-coloring of Type-I tree.

Proof (⇒) Follows directly from the Proposition 3.2, Proposition 3.4, Proposition 3.5.

(⇐) For a Type I tree let us prove that, $\chi_{g}(T)=5$. By the Proposition 3.2, Proposition 3.4, Proposition 3.5, $\chi_{g}(T)\geq 5$. To prove $\chi_{g}(T)\leq 5$, we show a graceful 5 - coloring of *T* using the Algorithm 3. Graceful coloring of a Type I tree is illustrated in the Fig. 2. □

**Algorithm 3:**
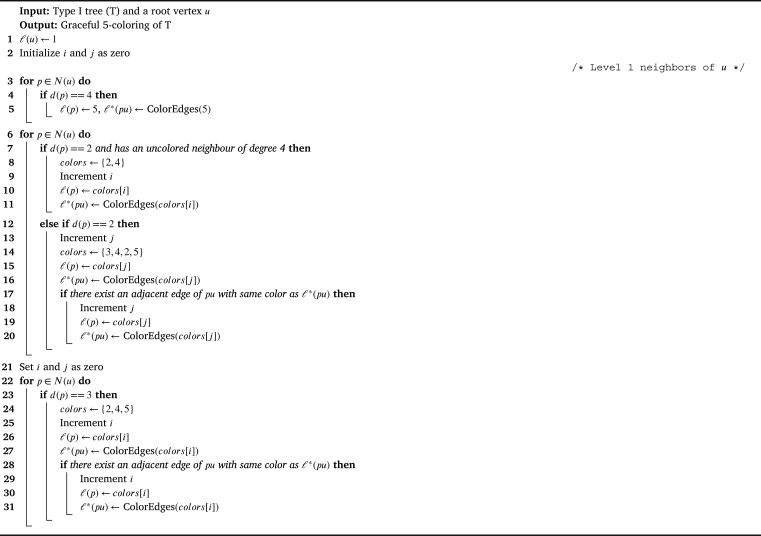
Graceful coloring of Type I trees (*T*,*u*).

**Algorithm 1:**
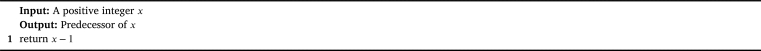
ColorEdges (*x*).

**Algorithm 2:**
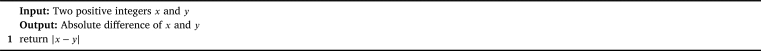
ColorAdjEdges (*x*,*y*).

**Figure:**
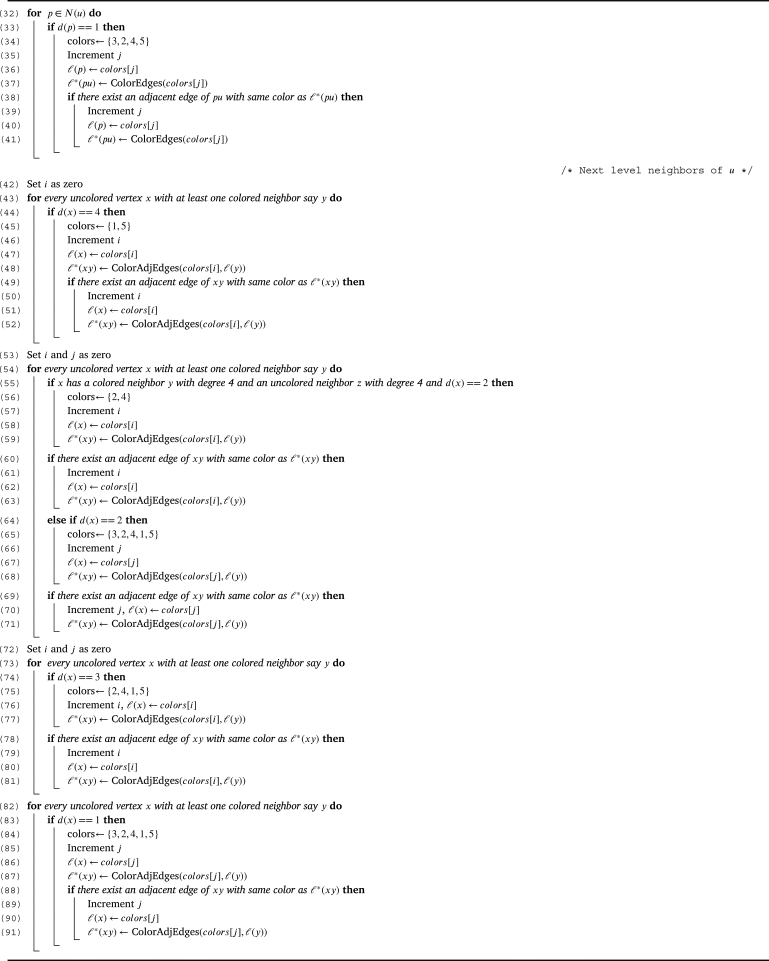

### 3.2 Type-II trees

Let Type II be the class of all trees in $T_{1}\cup T_{2}\cup T_{3}$ excluding the induced trees on $M_{T}$ and ${T_{3}}^{\prime }$.

Theorem 3.9 *For a tree*T∈T*,*$\chi_{g}(T)=6$*if and only if T is of Type II.*

**Figure 3:**
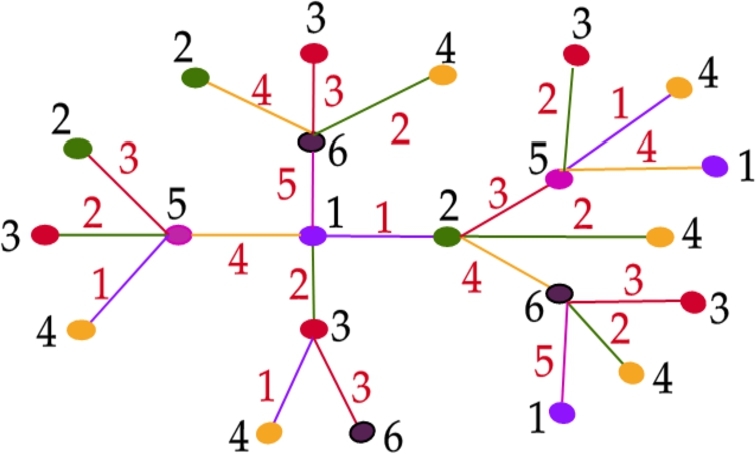
Graceful 6-coloring of Type-II tree.

Proof (⇒) Follows directly from the Proposition 3.2 and Proposition 3.7.

(⇐) For a Type II tree *T*, we shall prove that $\chi_{g}(T)=6$. By the Theorem 3.8 and Proposition 3.7, $\chi_{g}(T)\geq 6$. To prove $\chi_{g}(T)\leq 6$, we show a graceful 6 - coloring of *T* using the Algorithm 4. Graceful coloring of a Type II tree is illustrated in the Fig. 3. □

**Algorithm 4:**
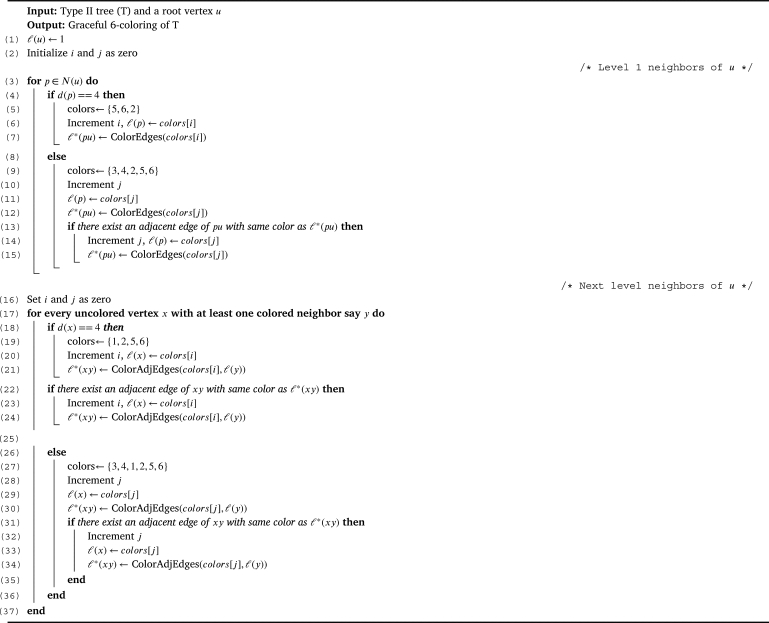
Graceful coloring of Type II trees (*T*,*u*).

### 3.3 Type-III trees

Let Type III be the class of all trees *T* in T which is either $T[M_{T}]$ or the trees induced by $T_{3}^{\prime }$ (or both).

Theorem 3.10 *For a tree*T∈T*,*$\chi_{g}(T)=7$*if and only if T is of Type III.*

**Figure 4:**
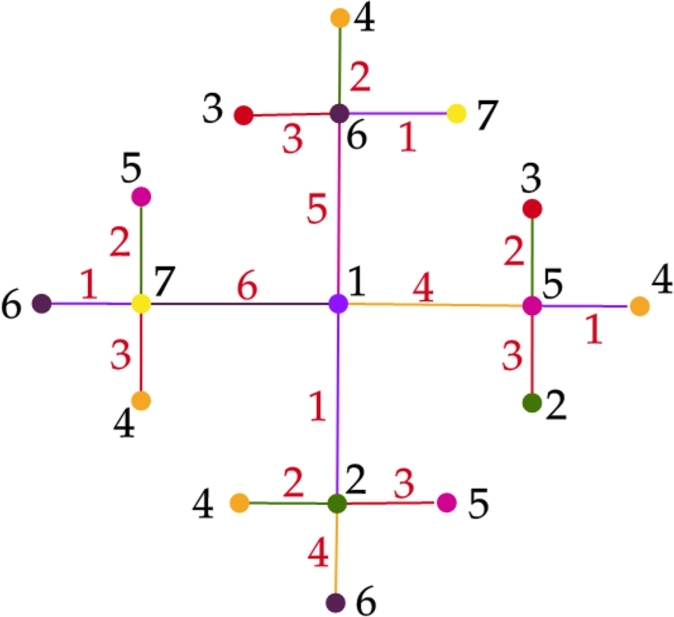
Graceful 7-coloring of Type-III tree.

Proof (⇒) Follows directly from the Theorem 3.8 and Theorem 3.9.

(⇐) For a Type III tree *T*, let us prove $\chi_{g}(T)=7$. From the Theorem 3.8 and Theorem 3.9 it is evident that, $\chi_{g}(T)\geq 7$. To prove the upper bound, we show a graceful 7 - coloring of *T* using the Algorithm 5. Graceful coloring of a Type III tree is illustrated in the Fig. 4. □

**Algorithm 5:**
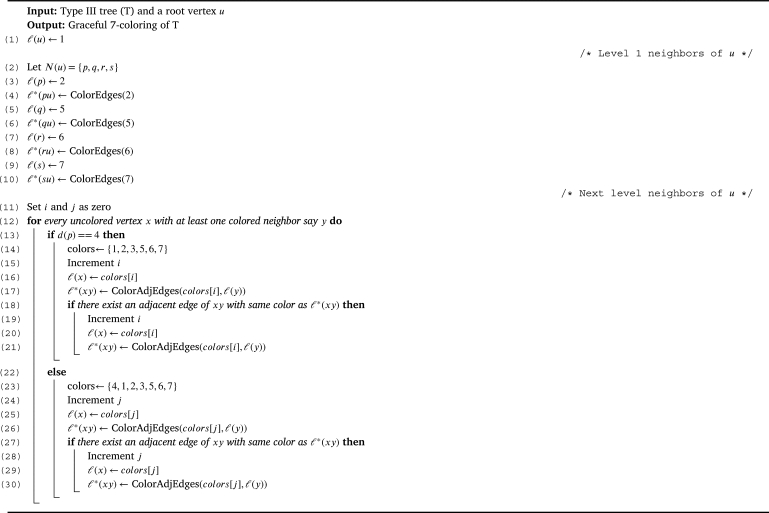
Graceful coloring of Type III trees (*T*,*u*).

## 4 Conclusion

The concept of graceful coloring of general classes of graphs are not much explored. Hence, we study the bounds for $\chi_{g}(T)$, where *T* is a tree with maximum degree as 4. It is also interesting and challenging to work on the following open problems:

Problem 1 Graceful chromatic number of bipartite graphs, complete graphs, split graphs, and complement of bipartite graphs.

Problem 2 Graceful chromatic number of various graph products, namely cartesian product, tensor product, corona product, etc.

## CRediT authorship contribution statement

**Devi Yamini S:** Conceived and designed the experiments; Performed the experiments; Analyzed and interpreted the data; Wrote the paper.

**Laavanya D:** Conceived and designed the experiments; Performed the experiments; Analyzed and interpreted the data; contributed reagents, materials, analysis tools or data; Wrote the paper.

## Declaration of Competing Interest

The authors declare that they have no known competing financial interests or personal relationships that could have appeared to influence the work reported in this paper.

## Data Availability

No data was used for the research described in the article.
